# Density Functional Theory-Based Calculation Shed New Light on the Bizarre Addition of Cysteine Thiol to Dopaquinone

**DOI:** 10.3390/ijms22031373

**Published:** 2021-01-29

**Authors:** Ryo Kishida, Shosuke Ito, Manickam Sugumaran, Ryan Lacdao Arevalo, Hiroshi Nakanishi, Hideaki Kasai

**Affiliations:** 1 Department of Biomaterials, Faculty of Dental Science, Kyushu University, Maidashi, Fukuoka 812-8582, Japan; 2 Institute for Melanin Chemistry, Fujita Health University, Toyoake, Aichi 470-1192, Japan; sito@fujita-hu.ac.jp; 3 Department of Biology, University of Massachusetts Boston, 100 Morrissey Boulevard, Boston, MA 02125-3393, USA; Manickam.Sugumaran@umb.edu; 4 Department of Physics, Talamban Campus, University of San Carlos, Cebu City 6000, Philippines; ryanlarevalo@gmail.com; 5 National Institute of Technology, Akashi College, Akashi, Hyogo 674-8501, Japan; nakanishi@akashi.ac.jp (H.N.); kasai@dyn.ap.eng.osaka-u.ac.jp (H.K.); 6 Institute of Industrial Science, The University of Tokyo, Meguro, Tokyo 153-8505, Japan; 7 Graduate School of Engineering, Osaka University, Suita, Osaka 565-0871, Japan

**Keywords:** dopaquinone, cysteine, melanin, density functional theory, quinone reactions, thiol addition to quinone

## Abstract

Two types of melanin pigments, brown to black eumelanin and yellow to reddish brown pheomelanin, are biosynthesized through a branched reaction, which is associated with the key intermediate dopaquinone (DQ). In the presence of l-cysteine, DQ immediately binds to the –SH group, resulting in the formation of cysteinyldopa necessary for the pheomelanin production. l-Cysteine prefers to bond with aromatic carbons adjacent to the carbonyl groups, namely C5 and C2. Surprisingly, this Michael addition takes place at 1,6-position of the C5 (and to some extent at C2) rather than usually expected 1,4-position. Such an anomaly on the reactivity necessitates an atomic-scale understanding of the binding mechanism. Using density functional theory-based calculations, we investigated the binding of l-cysteine thiolate (Cys–S^−^) to DQ. Interestingly, the C2–S bonded intermediate was less energetically stable than the C6–S bonded case. Furthermore, the most preferred Cys–S^−^-attacked intermediate is at the carbon-carbon bridge between the two carbonyls (C3–C4 bridge site) but not on the C5 site. This structure allows the Cys–S^−^ to migrate onto the adjacent C5 or C2 with small activation energies. Further simulation demonstrated a possible conversion pathway of the C5–S (and C2–S) intermediate into 5-*S*-cysteinyldopa (and 2-*S*-cysteinyldopa), which is the experimentally identified major (and minor) product. Based on the results, we propose that the binding of Cys–S^−^ to DQ proceeds via the following path: (i) coordination of Cys–S^−^ to C3–C4 bridge, (ii) migration of Cys–S^−^ to C5 (C2), (iii) proton rearrangement from cysteinyl –NH_3_^+^ to O4 (O3), and (iv) proton rearrangement from C5 (C2) to O3 (O4).

## 1. Introduction

Melanin, the polyphenolic pigment found throughout living organisms, is an important biopolymer that provides protection against damaging solar radiation [1,2,3,4,5,6,7,8,9]. In animals, specialized cells called melanocytes produce melanin pigments and transport them to the skin, hair, and eyes, where it provides external coloration. Understanding the biochemically distinct nature of melanocytes and melanocyte-related tissue reactions is crucial to treat diseases associated with melanogenic processes such as albinism, leukoderma, melanoma cancer, and other related skin disorders.

**Figure 1:**
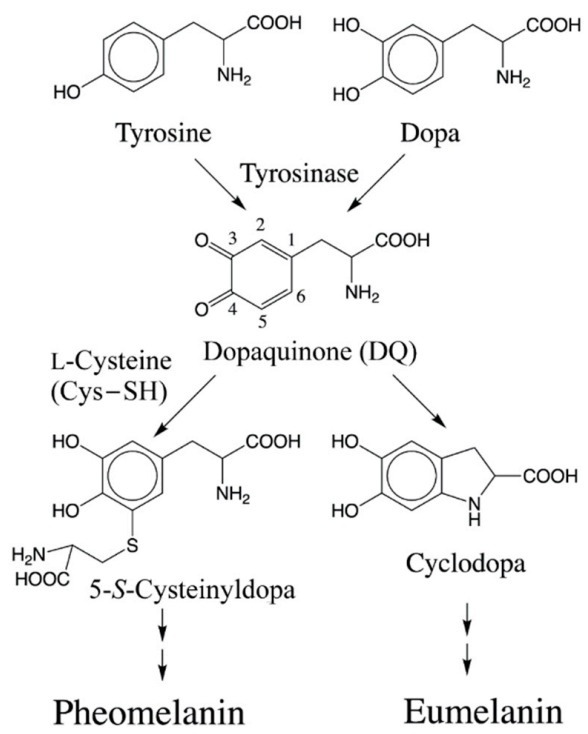
Initial stages of melanin biosynthesis. Tyrosine and its hydroxylated product dopa are converted to dopaquinone (DQ) by the enzyme, tyrosinase. Further reactivity of DQ determines the nature of melanin product formed. Non-enzymatic addition of Cys−SH results in the formation of 5-*S*-cysteinyldopa (or 2-*S*-cysteinyldopa) as the major (or minor) product. Oxidative polymerization of cysteinyldopa generates yellow to reddish brown pheomelanin product. On the other hand, intramolecular cyclization of DQ produces cyclodopa which leads to brown to black eumelanin.

Biosynthesis of melanin (melanogenesis) is initiated by tyrosinase-catalyzed oxidation of the amino acid, tyrosine and its hydroxylated dopa to dopaquinone (DQ). At DQ level, two important reactions determine the nature of melanin formed (Figure 1).

Interestingly, both reactions occur without any enzymatic assistance and are considered key examples of novel biological reactions that are of a non-enzymatic nature. In the presence of cellular thiols such as l-cysteine (Cys–SH), DQ exhibits rapid non-enzymatic addition. Thus, DQ and similar *o*-quinones bind to cellular thiols, including Cys–SH, glutathione, and protein thiols [1,2,3,4,9,10,11,12]. The binding of cellular Cys–SH to DQ produces cysteinyldopa which further transforms into yellow to reddish brown pheomelanin (pheomelanogenesis). After cellular Cys–SH is consumed enough (<1 μmol/L), DQ undergoes the competing reaction, i.e., intramolecular cyclization of the alanyl side chain [13]. This cyclization produces cyclodopa which after further transformations leads to brown to black eumelanin production (eumelanogenesis). Thus, melanin is a mixed pigment which consists of eumelanin and pheomelanin. The presence of pheomelanin influences the property of melanin in various contexts, including the color [14,15] and the binding ability for metals and/or drugs [16,17]. Furthermore, pheomelanogenesis can also cause cytotoxic and/or carcinogenic biochemical reactions [18,19,20,21,22]. 

Such non-enzymatic reactions are not limited to DQ alone as a number of other related catecholamine-derived quinones have also been shown to participate in a variety of biological processes. For example, neuromelanin production is associated with the non-enzymatic reactions of quinonoid products derived from dopamine [5]. Insects and other arthropods generate *N*-acyldopamine quinones as key components of sclerotized cuticle during the hardening of their exoskeleton [23]. Peptidyl dopa-derived quinonoid products are also established to play crucial role in mussel glue protein [24,25], as well as tunic formation and defense reaction in tunicates [26]. As a result, extensive studies have been carried out on the reactions of quinones with biological nucleophiles [4,7].

One of the most intriguing aspects of the reactivity of *o*-quinones with different nucleophiles is the fact that while most nucleophiles including amines exhibit normal Michael 1,4-addition reaction to quinones, thiols uniquely exhibit abnormal Michael 1,6-addition reactions. Moreover, in spite of the fact that proximity effects play a crucial role in tremendously accelerating the course of any chemical as well as biological reaction, the reaction of suitably situated internal amine group with the quinone ring in the same molecule is in fact slower than the reaction of externally present thiol group with a quinone. Such unusual reactivities of thiols have puzzled several organic chemists for decades.

Atomic-scale understanding of thiol binding to quinones could shed light on pheomelanogenesis and related processes. Effectiveness of density functional theory- (DFT-) based calculation for obtaining the potential energy hypersurface of amino acid motions as well as their electronic structures has been widely validated [27,28,29,30,31,32,33,34,35,36]. Previous computational studies revealed that thiolates undergo a charge transfer to *o*-quinones during the addition reaction [31,32]. Thus, thiols prefer lower levels of lowest unoccupied molecular orbital (LUMO) of *o*-quinones, thereby the electrons occupy the vacant orbitals with higher affinity. Furthermore, the cyclization of the alanyl side chain of DQ increases the LUMO level [31]. Therefore, the binding of thiols would become unfavorable for the cyclized products. This explains the reported competing behavior of *o*-quinones from the electronic point of view.

The addition reaction of Cys–SH derivatives to DQ derivatives has been investigated using several analysis techniques including high performance liquid chromatography (HPLC) [8,10,37,38], pulse radiolysis [9], and stopped-flow spectrophotometry [39] under enzymatic or electrochemical oxidation. This reaction proceeds through the binding of sulfhydryl (–SH) sulfur to an aromatic carbon [1,2,3,4,10]. The yield of 6-adduct (6-*S*-cysteinyldopa) has been reported to be only 1%, together with a relatively high amount of 5-adduct (74%) and 2-adduct (14%) [1,2,3,4,10], indicating the importance of the aromatic C5 and C2 carbon atoms. As a possible mechanism, the 1,6-Michael addition mechanism of Cys–SH has been proposed [37,38,39]. On the other hand, the competing nucleophilic reaction, i.e., cyclization of the alanyl side chain, occurs at C6 atom, corresponding to 1,4-Michael addition [40]. The reported positive correlations between pH and the addition rate indicate a base-catalyzed character of the reaction [38,39]. Thus, the binding of thiols would be initiated by deprotonation from the –SH group. 

While these experimental results provided insights into the Cys–SH reaction with DQ, it is imperative to establish a robust atomic-scale understanding of the mechanism of this reaction. In this current study, we investigated the binding mechanism of Cys–S^−^ to DQ using density functional theory-based first principles calculations. Briefly, results show quasi-stable Cys–S^−^-attacked intermediates with their binding sites at the C5, C2, C6, C3–C4 (bridge), and C1. Interestingly, the C2–S bonded intermediate was found to be less energetically stable than the C6–S bonded case. The most preferred Cys–S^−^-attacked intermediate is at the carbon-carbon bridge between the two carbonyls (C3–C4 bridge) but not at C5. This structure allows the Cys–S^−^ to migrate onto the adjacent C5 or C2 with small activation energies. Further simulation demonstrated a possible conversion pathway of the C5–S (and C2–S) intermediate into 5-*S*-cysteinyldopa (and 2-*S*-cysteinyldopa), which is the experimentally identified major (and minor) product.

## 2. Results 

### 2.1. Initial Binding Sites for Cysteine Thiolate (Cys−S^−^) on Dopaquinone (DQ) 

**Figure 2:**
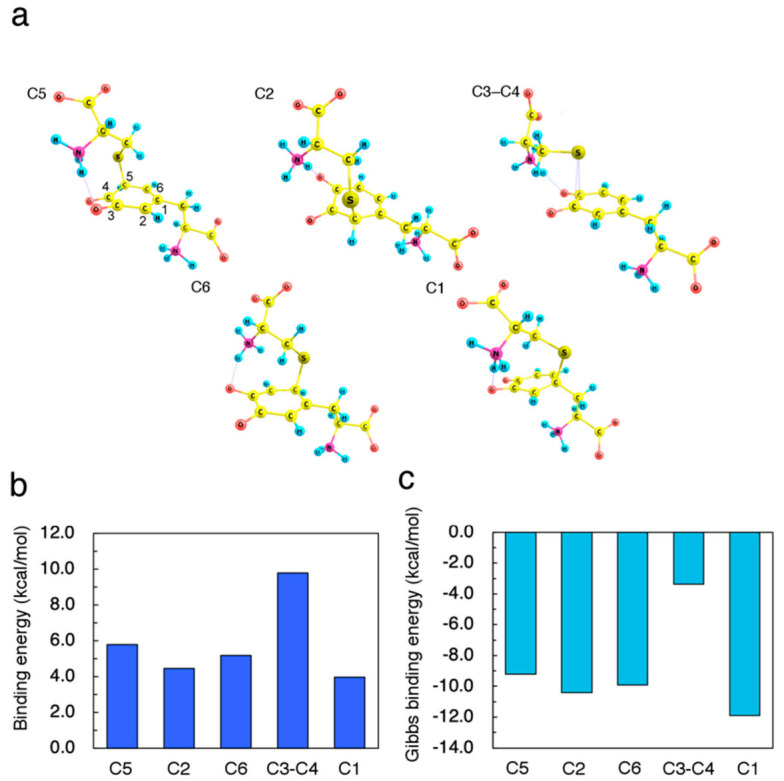
Properties of Cys–S^−^-attacked dopaquinone (DQ) intermediates. (**a**) Optimized geometries resulting from (C5) 5-addition, (C2) 2-addition, (C6) 6-addition, (C3–C4) bound on 3,4-bridge, and (C1) 1-addition. (**b**) Binding energies *E*_b_ of Cys–S^−^ on DQ, which are defined as *E*_b_ = (*E*_Cys–S_^−^ + *E*_DQ_) − *E*_Cys–S_^−^_–DQ_, where *E*_Cys–S_^−^, *E*_DQ_, and *E*_Cys–S_^−^_–DQ_ are the total energy of Cys–S^−^, DQ, and Cys–S^−^-attacked DQ, respectively. (**c**) Gibbs binding energies of Cys–S^−^ on DQ computed based on the vibrational analysis at 37.0 °C.

To identify the initial process of cysteine binding, we investigated the energetic preference of thiolate-attacked intermediates. We performed geometrical optimization of DQ with Cys−S^−^ located around the benzene ring. We found five binding sites, namely C5, C2, C6, C3–C4 (bridge), and C1. Conformational rotation of DQ and Cys−S^−^ gave various isomers with slightly different binding energies. Moreover, hydrogen bonding between DQ and Cys−S^−^ also resulted in the formation of various bound states. For simplicity, we present here the bound structures based on the energetically favorable conformation in the isolated state, and focus on the most stable hydrogen-bonded structures. The optimized structures, the binding energies, and the Gibbs binding energies (based on vibrational analyses) are shown in Figure 2a–c, respectively.

The vibrational analysis for the Cys–S^−^-attacked intermediates confirmed no imaginary frequencies. As shown in Figure 2, the C2-bound structure was less energetically favorable than the C6-bound case. Furthermore, Cys−S^−^ preferred the C3−C4 bridge site more than C5 site as an immediate active site. The negative values of the Gibbs binding energies indicate that the Cys–S^−^-attacked intermediates themselves cannot be the thermodynamically stable products. Therefore, further conversion pathway must be explored.

**Figure 3:**
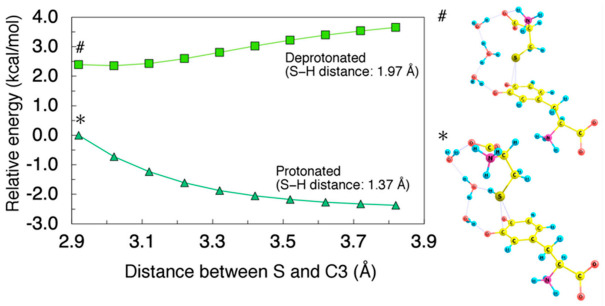
(**Left**) Minimum energy path for Cys–S^−^ (S–H distance: 1.97 Å) and Cys–SH (S–H distance: 1.37 Å) coordination on C3−C4 bridge plotted as squares and triangles, respectively. H_2_O trimer was used as an acceptor for dissociating proton. Energies were referenced to the total energy of the protonated Cys–SH-DQ complex with 2.92 Å of C3−S distance. All geometrical parameters except for the C3−S and S−H distance were allowed to relax. (**Right**) Cys–S^−^- (#) and Cys–SH- (*) coordinated structure.

The non-deprotonated cysteine (Cys−SH) did not show any binding ability to DQ, as manifested by spontaneous dissociation upon geometrical optimization. To demonstrate the instability of Cys−SH-attacked intermediates, we calculated the minimum energy paths for the Cys−S^−^ and Cys−SH coordination on the C3−C4 bridge as shown in Figure 3. To directly compare the protonated and deprotonated cases, we considered H_2_O trimer as an acceptor for the dissociated proton. The obtained energy profiles clearly show that the interaction between the protonated Cys−SH and DQ is repulsive while the deprotonated Cys−S^−^ can be attracted by DQ.

### 2.2. Migration of Cysteine Thiolate (Cys−S^−^)

**Figure 4:**
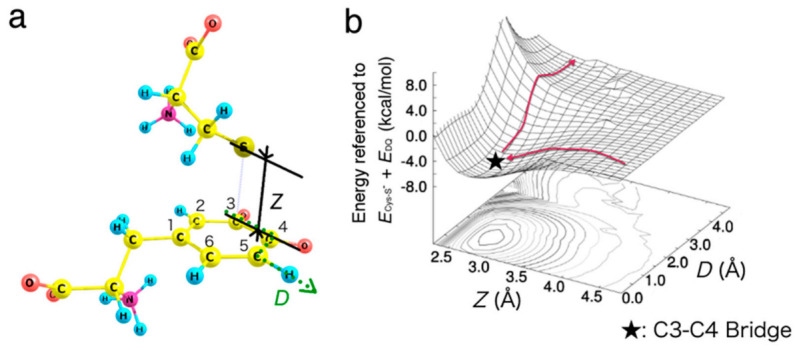
Migration of Cys−S^−^ on the benzene ring of DQ. (**a**) Interaction model between Cys–S^−^ and DQ with two variables, *Z* and *D*. (**b**) Potential energy surface for the approaching/migration of Cys–S^−^ on DQ around C3–C4 and C5. Energies are referenced to the sum of total energy of isolated systems, i.e., Cys–S^−^ and DQ (*E*_Cys–S_^−^ + *E*_DQ_). The benzene ring carbon atoms, and all the Cys−S^−^ atoms were fixed, and the other degrees of freedom were relaxed.

Based on the intermediate structures obtained, we next investigated the migration of Cys−S^−^ on the benzene ring of DQ. Although C3−C4 bridge was identified as the most stable binding site, the final product would be the C5- or C2-adduct. Thus, the bound state on C3−C4 must be connected to that on C5 or C2. In order to describe the interaction between DQ and Cys−S^−^, we considered two coordinates *Z* and *D*, as defined in Figure 4a.

*Z* is the height of Cys−S^−^ as measured from C3, and *D* increases as Cys−S^−^ migrates along the perimeter of the benzene ring. The calculated potential energy surface along the two degrees of freedom *Z* and *D* is shown in Figure 4b. Note that the benzene ring carbon atoms, and all the Cys−S^−^ atoms were fixed, and the other degrees of freedom were relaxed during the calculation. Our result shows the absence of activation barrier for the binding onto C3−C4 bridge, while the potential energy increases at around the C5 site.

**Figure 5:**
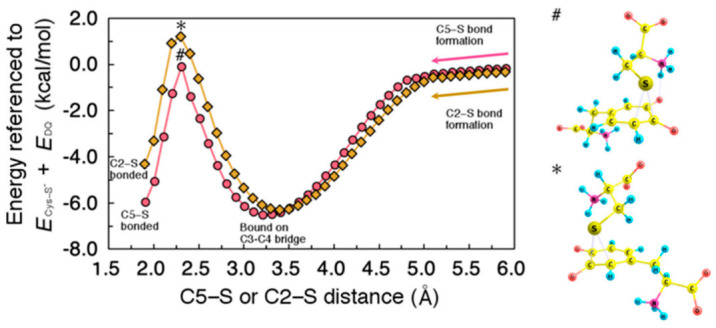
(**Left**) Minimum energy path for the migration of Cys–S^−^ on 5-carbon (C5) and 2-carbon (C2), plotted as circles and diamonds, respectively. Energies were referenced to the sum of total energy of isolated systems, i.e., Cys–S^−^ and DQ (*E*_Cys-S_^−^ + *E*_DQ_). All geometrical parameters except for C5–S or C2–S distance were allowed to relax. (**Right**) Transition state structure for C5–S (#) and C2–S (*) bond formation.

Although the potential energy surface calculated provides an overview of the initial phase of reaction, it was not able to find a stable C5-bound state potentially due to the fixed carbon atoms. Therefore, we further performed calculations to obtain the minimum energy paths with completely relaxed coordinates except for the designated C5−S or C2−S bond length. The obtained minimum energy paths are shown in Figure 5.

Note that these calculations were carried out along the direction of C5−S or C2−S dissociation (i.e., from the left to the right in Figure 5), although the cysteine binding proceeds in the opposite direction. As shown in Figure 5, Cys−S^−^ is initially bound onto C3−C4 bridge, and then can migrate to C5 or C2 with a moderately small activation energy. The migration barrier to C2 (7.5 kcal/mol) is slightly higher than that to C5 (6.4 kcal/mol), indicating the preference of C5-bound state. Note also that the C3−C4-bound states appeared in the minimum energy paths were not hydrogen-bonded between DQ and Cys−S^−^, thereby showing weaker binding as compared to the ones shown in Figure 2. 

In the calculations for the minimum energy paths, we tried to provide a smooth connection between the binding sites. Our calculations did not find reaction paths keeping hydrogen-bonded throughout the migration mainly due to the conformational limitations. Therefore, we present the migration paths that start from where the hydrogen bond is absent. Although not explicitly discussed here, the hydrogen-bonded state at the C3-C4 can also be involved in the reaction, and can be easily switched to non-hydrogen-bonded state for the subsequent migration to the C5 or C2 at lower energy cost (3.4 kcal/mol).

### 2.3. Proton Rearrangements to Give Cysteinyldopa

**Figure 6:**
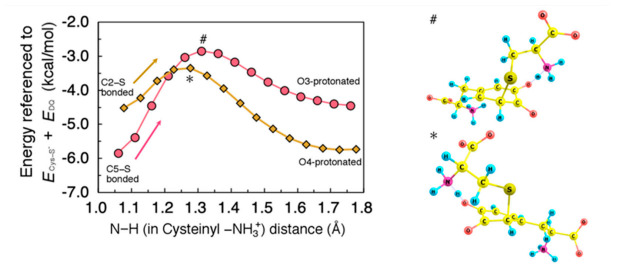
(**Left**) Minimum energy path for proton rearrangement from cysteinyl −NH_3_ group in Cys–S^−^ to 3-oxygen (O3) after C5–S bond formation, and 4-oxygen (O4) after C2–S bond formation, plotted as circles and diamonds, respectively. Energies were referenced to the sum of total energy of isolated systems, i.e., Cys–S^−^ and DQ (*E*_Cys-S_^−^ + *E*_DQ_). All geometrical parameters except for the N–H distance were allowed to relax. (**Right**) Transition state structure for O3- (#) and O4- (*) protonation.

Finally, we investigated the processes to form the product 5-*S*-cysteinyldopa (and 2-*S*-cysteinyldopa) from the C5−S (and C2−S) bonded intermediate structure. For this conversion, the quinonic oxygens O3 and O4 must be both protonated, and the attacked C5 (C2) needs to be deprotonated. As shown in Figure 6, the C5−S (C2−S) bonded intermediate can form hydrogen bonding between cysteinyl –NH_3_^+^ and O3 (O4). 

Therefore, it is straightforward to consider that proton transfer initially occurs between this pair of hydrogen-bonded groups. Figure 6 shows the minimum energy path along proton transfer from cysteinyl –NH_3_^+^ to O3 (O4). The activation barrier for the O3−(O4−) protonation from –NH_3_^+^ was estimated to be 3.0 (1.2) kcal/mol, indicating that this process is not a rate-determining step.

**Figure 7:**
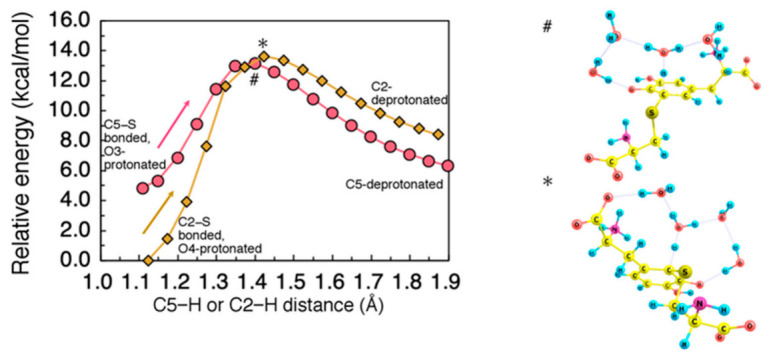
(**Left**) Minimum energy path for proton dissociation from C5 after −NH_3_-to-O3 proton rearrangement, and from C2 after −NH_3_-to-O4 proton rearrangement. H_2_O tetramer was used as an acceptor for dissociating proton. Energies were referenced to the total energy of the initial state structure for C2-deprotonation. All geometrical parameters except for the C5−H or C2−H distance were allowed to relax. (**Right**) Transition state structure for C5- (#) and C2- (*) deprotonation.

The proton transfer to O3 (O4) can be followed by proton rearrangement from C5 (C2) to O4 (O3). As a possible proton acceptor that mediates this rearrangement, we considered H_2_O tetramer interacting with the Cys−S^−^-DQ system. As in Grotthus mechanism, a hydrogen-bonded H_2_O network effectively stabilizes the dissociated proton and enables efficient proton diffusion. Using this H_2_O tetramer, we calculated the minimum energy path along C5− (C2−) deprotonation. The obtained energy profile is shown in Figure 7. The activation barrier for the C5− (C2−) deprotonation was estimated to be 8.4 (13.6) kcal/mol.

## 3. Discussion

### 3.1. Structural and Electronic Analyses for the Initial Intermediates

In this study, we identified that the most stable initial intermediate of the Cys−S^−^-DQ system is when Cys−S^−^ is at the C3–C4 bridge rather than at the C5 site that was expected from the previous experiments [1,2,3,4,10].

Essential geometrical parameters of the Cys–S^−^-attacked intermediates are listed in Table 1. The C3−C4-bound structure has a relatively long C−S bond length (2.75 Å of C3−S bond length), indicating a less ordinary covalent bonding unlike the other cases; for instance, the C5-bound structure shows 1.91 Å of C5−S bond length. The unusual covalent nature of the C3−C4-bound structure also manifests in a relatively small geometrical alteration upon binding unlike the other cases exhibiting a *sp*^2^-to-*sp*^3^ (planar-to-pyramidal) structural change during the Cys–S^−^-attack as shown in Table 1.

As representative electronic state characterization, Table 2 lists the natural charges on S (in Cys–S^−^), O3, and O4 for the Cys–S^−^-attacked intermediates, which were obtained through the natural population analysis (NPA). Consistent with our previous study [31,32], the analysis clearly demonstrates an electronic charge transfer from cysteinyl sulfur to quinonic oxygens. The C3−C4-bound intermediate presenting a planar geometry of quinone showed a moderate change in the charged state, which is milder than the other cases showing more drastic amounts of charge transfer.

The binding of cysteine to dopaquinone is a fairly rapid process that takes place with a rate constant of 3 × 10^7^ L mol^−1^ s^−1^ at lower concentrations of Cys−SH and at neutral pH [13]. Even though advanced techniques such as stopped-flow spectrophotometry, flash photolysis, and pulse radiolysis, have enabled ultrafast time-resolved spectroscopy, the presence of C3−C4-bound states has not been found. Integrating the above analyses, the C3−C4-bound intermediate can be said to be both structurally and electronically less perturbed from the isolated state, potentially contributing to the absence of activation barrier for Cys–S^−^ attack, as demonstrated in Figure 4 and Figure 5. In other words, due to this nature, the C3−C4-bound intermediate might not give easy detectable responses upon typical spectroscopic perturbations, making experimental identifications difficult. The presence of such an energy landscape, that enables reactions to occur at nearly zero energy cost, may explain the reason why Cys−SH but not the other non-thiolic cellular compounds can compete the rapid cyclization of DQ.

Throughout this study, we used the Becke’s three-parameters hybrid functional [41] combined with the Lee-Yang-Parr correlation functionals [42] (B3LYP) as the exchange-correlation potential. This exchange correlation functional has been widely successful in describing typical organic chemical reactions as well as electron densities. Nevertheless, in several cases B3LYP fails to accurately account for non-covalent interactions due to London dispersion force. Considering its non-local nature, the accurate calculation of such non-covalent interactions may be improved by increasing the weight of exact exchange potential and including meta-GGA functionals. As mentioned above, we found a non-covalent nature of the C3−C4-bound intermediate, which is energetically more stable than the covalent-bonded C5-bound intermediate. In order to assess the validity of B3LYP functional, we compared the binding energies at the C3−C4 bridge and C5 site using different exchange correlation functionals as representative results. For this comparison, we used mPW1PW91 [43], M06-2X [44], and CAM-B3LYP [45] for the structural optimization as well as the total energy calculation. The increased weight of exact exchange potential and the inclusion of a meta-GGA functional resulted in higher binding energies regardless of the binding sites (Table S1). However, the choice of exchange correlation functional did not remarkably affect the energetic preference. In other words, B3LYP functional can be regarded as sufficient for elucidating the cysteine addition reaction.

As pointed out by previous studies [38,39], cysteine binding is a base-catalyzed nucleophilic addition reaction. This is consistent with our results where protonated Cys–SH (but not Cys–S^−^) did not show any binding ability to DQ as demonstrated in Figure 3. As the initial step of the reaction, a previous study assumed an equilibrium of cysteine protonation/deprotonation in the presence of DQ to propose a kinetic model [39]. However, it was not clarified whether the deprotonation occurs before or during the binding to DQ. Based on the potential energy curves shown in Figure 3, it is more plausible that cysteine undergoes deprotonation prior to the binding with DQ.

Although we assumed cysteinyl deprotonation as the initial step, an alternate free radical-mediated addition was also proposed to account for the abnormal addition of thiols to *o*-quinones [46]. This mechanism exploits the reducing property of thiols to account for the observed Michael 1,6-addition product. According to this mechanism (Table 3, Figure S1), thiols initially reduce the *o*-quinone to semiquinone by one electron transfer reaction.

The rapid coupling of the resultant semiquinone with the thiyl radical would produce the unconventional 1,6-adduct and not the typical Michael 1,4-adduct. Correspondingly, in triplet state, there were no bound states for the C3–C4- and the C6-bound structure, while the C5-bound structure exhibited a quasi-stable bound state, as shown in Table 3. However, all the radical species calculated were less stable than the corresponding spin-singlet system. Further detailed studies for possible redox reactions between Cys–SH and DQ are needed to unveil the reaction to the generalized extent of more oxidative conditions.

### 3.2. Effects of Cysteinyl Amino Group on Binding Sites and Reaction Rate

The preference of 1,6-Michael addition over 1,4-Michael addition can be explained by considering the presence of the C3–C4-bound state, which is formed with high affinity and without activation energy. As mentioned, the C3–C4 bridge acts as an immediate binding site for selective C5- and C2-binding by allowing Cys–S^−^ to migrate to the adjacent sites. Therefore, the energetic stability of the C3–C4-bound state is one of the most important factors contributing to the selective formation of 5-adduct and 2-adduct. 

The stability of the C3–C4-bound state is also partially derived from the hydrogen bond between cysteinyl –NH_3_^+^ and DQ. In fact, this hydrogen bonding on C3–C4 was stronger than that on C6, as shown in Table 4. In other words, the relative energetic stability on C3–C4 with respect to that on C6 could become less significant without hydrogen bonding. Therefore, a thiol lacking primary amino groups, such as glutathione, *N*-acetylcysteine, and thioglycolic acid, would exhibit an increased yield of 6-adduct due to the absence of hydrogen bonds. In fact, glutathione was reported to react with DQ to give 76, 12 and 5% yields of 5-*S*-glutathionyldopa, 2-*S*-glutathionyldopa, and 6-*S*-glutathionyldopa, respectively [47]. The yield of 6-adduct for glutathione addition (5%) is higher than that for cysteine addition (1%) [1,2,3,4,10]. Thus, although still minor, an amino-free thiol would possess slightly reactive C6, thereby affecting the structure of pheomelanin produced.

Pheomelanin is a pigment consisting of benzothiazines and benzothiazoles as building monomers [48]. After the formation of 5-*S*-cysteinyldopa and 2-*S*-cysteinyldopa, they undergo redox exchange with unreacted DQ, and then cyclize to form quinone imines through the cysteinyl –NH_3_^+^ and the quinonic carbonyl [48]. This cyclization is a necessary process for the further conversion to benzothiazines and benzothiazoles. On the other hand, 6-*S*-cysteinyldopa cannot produce such cyclized benzothiazine chromophores, considering the distance between the cysteinyl –NH_3_^+^ and the quinonic carbonyl. Therefore, the preference of 1,6-Michael addition over 1,4-Michael addition is of great significance in determining the color of pheomelanin.

Cysteinyl –NH_3_^+^ also acts as a proton donor that enables rapid quinonic protonation with the very small activation energy. Therefore, we assumed that C5- (C2-) deprotonation occurs after O3- (O4-) protonation. On the other hand, our mechanism might not be directly extrapolated to thiols lacking proton donors such as primary amino groups. If a thiol does not have amino groups, the quinonic protonation becomes a diffusion-controlled process, thus deprotonation from the thiolate-attacked carbon atom may occur even before the quinonic protonation. As shown in Figure S2, we confirmed that the activation barrier for C5- (C2-) deprotonation becomes higher when O3 (O4) is not protonated, indicating slower reaction rate of amino-free thiols.

This view is consistent with a previous kinetic study on the thiol binding reactions, where the effect of the presence of cysteinyl –NH_3_^+^ was discussed by comparing with the amino-free analogue thioglycolic acid [39]. According to the kinetic analysis, the thioglycolic acid-attacked intermediate lacking amino groups is more stable, leading to a slower reaction rate because of the rate-limiting proton rearrangement.

### 3.3. Energy Diagram for Cysteine Binding to Form Cysteinyldopa

**Figure 8:**
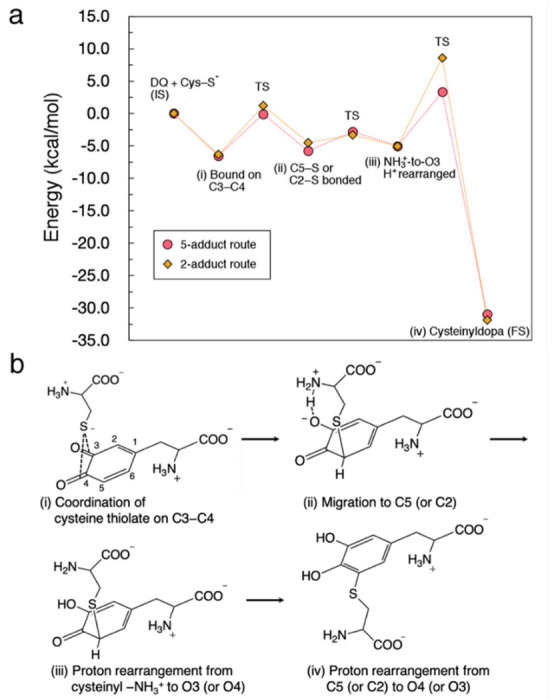
Overview of the reaction between Cys–S^−^ and DQ. (**a**) Energy diagram for the formation of 5-*S*-cysteinyldopa and 2-*S*-cysteinyldopa plotted as circles and diamonds, respectively. (**b**) Proposed binding mechanism.

To see the whole picture of cysteine binding, we show an energy diagram for the reaction to form 5-*S*-cysteinyldopa and 2-*S*-cysteinyldopa along with a hypothetical reaction scheme. Based on the geometrical optimization for each step and the potential energy curves, we estimated the energy change as shown in Figure 8a. In the final proton rearrangement, the Cys−S^−^-DQ system undergoes a significant stabilization, that makes this binding irreversible. A remarkable difference between 5-adduct and 2-adduct formation can be seen in the activation barrier for the final proton rearrangement [denoted as (iv)]. Therefore, the experimentally observed preference toward 5-*S*-cysteinyldopa would have originated from this elementary process. The corresponding hypothetical reaction scheme is shown in Figure 8b. Briefly, we propose that the binding of Cys–S^−^ to DQ proceeds with (i) coordination of Cys–S^−^ to C3–C4 bridge, (ii) migration of Cys–S^−^ to C5 (C2), (iii) proton rearrangement from cysteinyl –NH_3_^+^ to O4 (O3), and (iv) proton rearrangement from C5 (C2) to O3 (O4).

## 4. Materials and Methods 

### 4.1. Electronic State Calculation Methods

Throughout this work, we performed first-principles calculations based on DFT [49,50] using the Gaussian 09 computational package [51]. We used B3LYP functional as the exchange-correlation potential [41,42]. The calculations employed 6-31++G(d,p) basis set to expand the Kohn-Sham orbitals.

We estimated the atomic charges through the natural population analysis (NPA) [52,53]. As a solvation model, we considered dielectric response of surrounding water molecules by the integral equation formalism polarizable continuum model (IEF-PCM) [54]. We used the IEF-PCM for both the single point calculations and the structural optimizations. We carried out vibrational frequency analyses on the same level of theory for all the optimized structures to confirm their stability and to calculate the Gibbs free energies. For the Gibbs free energy calculations, we considered the degrees of freedom for the molecular vibration, rotation, and translation. The temperature and pressure were set to 37.0 °C and 1.0 atm, respectively. This thermodynamic model assumes a non-interacting ideal gas of DQ and Cys−S^−^ (with PCM correction), and that the pressure–volume product is uniquely determined by the temperature.

### 4.2. Structures for Calculations

Total energy changes during the reaction can be slightly affected by the choice of isomer. DQ and Cys−S^−^ includes a saturated hydrocarbon chain, that can rotate at a relatively lower energy cost. Furthermore, the presence of electrically polarized hydrogen-containing groups such as amino and carboxyl groups can easily be a cause of hydrogen bonding. Thus, conformational rotation and hydrogen bonding give various isomers with slightly different binding energies. For simplicity, here we constructed bound structures based on the energetically favorable conformation in the isolated state, and focused on the most stable hydrogen-bonded structures. Throughout the calculation, we used a consistent conformation of DQ and Cys−S^−^, although the hydrogen bonding site can switch at cysteinyl migration on DQ.

### 4.3. Reaction Analyses

The binding energy (*E*_b_) for Cys–S^−^ was defined as *E*_b_ = (*E*_Cys-S_^−^ + *E*_DQ_) − *E*_Cys-S-DQ_, where *E*_Cys-S_^−^, *E*_DQ_, and *E*_Cys-S-DQ_ are the total energy of Cys–S^−^, DQ, and Cys–S^−^-attacked DQ, respectively. A positive value of binding energy means an energetically favorable binding. The Gibbs binding energy was defined in the same manner.

Potential energy surfaces were calculated at specified points of geometry. For each point, partial geometrical optimization was conducted with some degrees of freedom held fixed (The detailed specification of the active and the frozen coordinates are given in the text). The potential energy surfaces were calculated so as to provide smooth motions of molecules. However, to precisely determine the transition state energy and structure, it is necessary to be optimized so that the structure exhibits only one imaginary frequency. In this study, our attempts to obtain the true transition states based on the vibration analysis were unsuccessful potentially due to the relatively flat nature of potential energy surface as well as the complexity of the internal motion. In other words, the reaction paths shown in this study are not fully parallel with the true intrinsic reaction coordinates. Therefore, the activation barriers shown in this study must be slightly overestimated.

Deprotonation reactions were described using H_2_O trimer or tetramer as a proton acceptor. The H_2_O trimer and tetramer were constructed based on a tetragonal hydrogen-bonded network structure, and then placed around the proton to be dissociated.

## 5. Conclusions

In the present study, we investigated the binding mechanism of l-cysteine to DQ using density functional theory-based calculation. We calculated the binding energies of Cys−S^−^-attacked intermediates and the minimum energy paths for the approach/migration of Cys−S^−^ on the aromatic carbons. We identified the C3−C4 bridge of DQ as the most preferable site for Cys−S^−^, while the protonated Cys−SH did not show binding ability at any binding sites of DQ. We found that the calculated minimum energy paths for the C5−S and C2−S bond formation involve a precursor Cys−S^−^-bound state on C3−C4 bridge. Therefore, the C5− and C2−S bond formation can be affected by this precursor state, causing moderately small activation barriers. The C5− and C2−S bond formation are followed by further proton rearrangement to form 5- and 2-S-cysteinyldopa, which are the major and minor products, respectively.

Based on our results, we propose that the binding of Cys−S- to DQ proceeds in the following sequence: (i) coordination of Cys−S- to C3−C4 bridge and (ii) migration of Cys−S- to C5 (or C2), (iii) proton rearrangement from cysteinyl –NH3+ to O3 (O4), and (iv) proton rearrangement from C5 (C2) to O4 (O3). Throughout the reaction, a significant stabilization occurs at the final step (iv), making the binding of cysteine irreversible.

The obtained findings in this study provide a foundation for understanding the mechanism of cysteine binding, and can be a basis for pheomelanogenesis.

## Figures and Tables

**Table 1:** C−S bond lengths and quinonic dihedral angles of Cys–S^−^-attacked intermediates.

Binding Site ^a^	C−S Bond Length (Å) ^b^	O3-C3-C4-O4 Dihedral Angle (Deg.) ^c^
Before reaction	N/A	0.27
C5	1.91	25.59
C2	1.92	25.19
C6	1.95	10.44
C3−C4	2.75	4.05
C1	1.96	13.33

^a^ As mentioned in Section 2.1., all the structures are chosen based on the most preferable conformation in the isolated state, and on the energetically most stable hydrogen bonding. ^b^ C5−S, C2−S, C6−S, C3−S, and C1−S are listed for the intermediates resulting from C5-, C2-, C6-, C3-C4-, and C1-attack of Cys–S^−^, respectively. ^c^ Dihedral angle is defined by relative rotation between O3 and O4 along C3−C4 axis. The origin of this dihedral angle was set to the completely planar structure.

**Table 2:** Natural charges on cysteinyl sulfur and quinonic oxygens during the formation of Cys–S^−^-DQ complex.

Binding Site	Natural Charges (e)		
	S	O3	O4
Before reaction	−0.73	−0.54	−0.53
C5	0.19	−0.80	−0.61
C2	0.19	−0.63	−0.81
C6	0.15	−0.63	−0.83
C3–C4	−0.28	−0.65	−0.70
C1	0.15	−0.82	−0.62

**Table 3:** Reaction energy for radical coupling reaction.

Binding Site	Reaction ^a^	Relative Energy (kcal/mol) ^b^
C5	Cys−SH + DQ → Cys−S· + DQ−H· (at O3)	12.9
	Cys−S· + DQ−H· → Cys−S·/DQ−H·	20.9
	Cys−S·/DQ−H· → Cys−S/DQ−H	−5.0
C3−C4	Cys−SH + DQ → Cys−S· + DQ−H· (at O3)	12.9
	Cys−S· + DQ−H· → Cys−S·/DQ−H·	Not bonded ^c^
	Cys−S·/DQ−H· → Cys−S/DQ−H	−9.2
C6	Cys−SH + DQ → Cys−S· + DQ−H· (at O4)	13.4
	Cys−S· + DQ−H· → Cys−S·/DQ−H·	Not bonded ^c^
	Cys−S·/DQ−H· → Cys−S/DQ−H	−12.9

^a^ Cys−S and DQ−H· respectively denote the thiyl radical and the semiquinone radical resulting from hydrogen atom transfer. The radical species were calculated by specifying doublet spin multiplicity. O3 was chosen as a binding site for hydrogen atom. Cys−S·/DQ−H·and Cys−S/DQ−H respectively denotes the cysteine-attacked intermediate in triplet and singlet state. ^b^ Relative energies were referenced to the total energy of the isolated system of Cys−SH and DQ. ^c^ Spontaneous dissociation upon structural optimization was observed. Initially, the corresponding singlet structure was optimized, and then re-optimized for the triplet state, resulting in the spontaneous dissociation.

**Table 4:** Effect of hydrogen bonding between cysteinyl –NH_3_^+^ and DQ on binding energy.

Binding Site	*ΔE*_b_ = *E*_b, Not HB_ − *E*_b, HB_ (kcal/mol) ^a^
C5	2.8
C2	4.6
C6	1.0
C3–C4	3.4
C1	4.5

^a^ The binding energy of non-hydrogen-bonded intermediate (*E*_b, Not HB_) was subtracted by that of hydrogen-bonded intermediate (*E*_b, HB_).

## Data Availability

The data presented in this study are available in this article and its accompanying supplementary materials.

## Supplementary Materials

The following are available online at https://www.mdpi.com/1422-0067/22/3/1373/s1, Figure S1: Alternate mechanism to account for the abnormal addition of thiols to quinone, Table S1: Comparison of binding energies at C5 and C3−C4 using different exchange correlation func-tionals.

## Abbreviations

Cys–SH	l-cysteine
DFT	density functional theory
DQ	dopaquinone
HPLC	high performance liquid chromatography
IEF-PCM	integral equation formalism polarizable continuum model
LUMO	lowest unoccupied molecular orbital
NPA	natural population analysis
