# Supplementary material for: Density Functional Theory-Based Calculation Shed New Light on the Bizarre Addition of Cysteine Thiol to Dopaquinone
Source: Int J Mol Sci. 2021 Jan 29;22(3):1373. doi: 10.3390/ijms22031373 (PMC7866380; doi:10.3390/ijms22031373)
Supplement: Supplementary file 1 [file ijms-22-01373-s001.pdf]

Supplementary Materials

Density Functional Theory-based Calculation Shed New Light on The Bizarre Addition of Cysteine Thiol to Dopaquinone.

Ryo Kishida, Shosuke Ito, Manickam Sugumaran, Ryan Lacdao Arevalo, Hiroshi Nakanishi, Hideaki Kasai

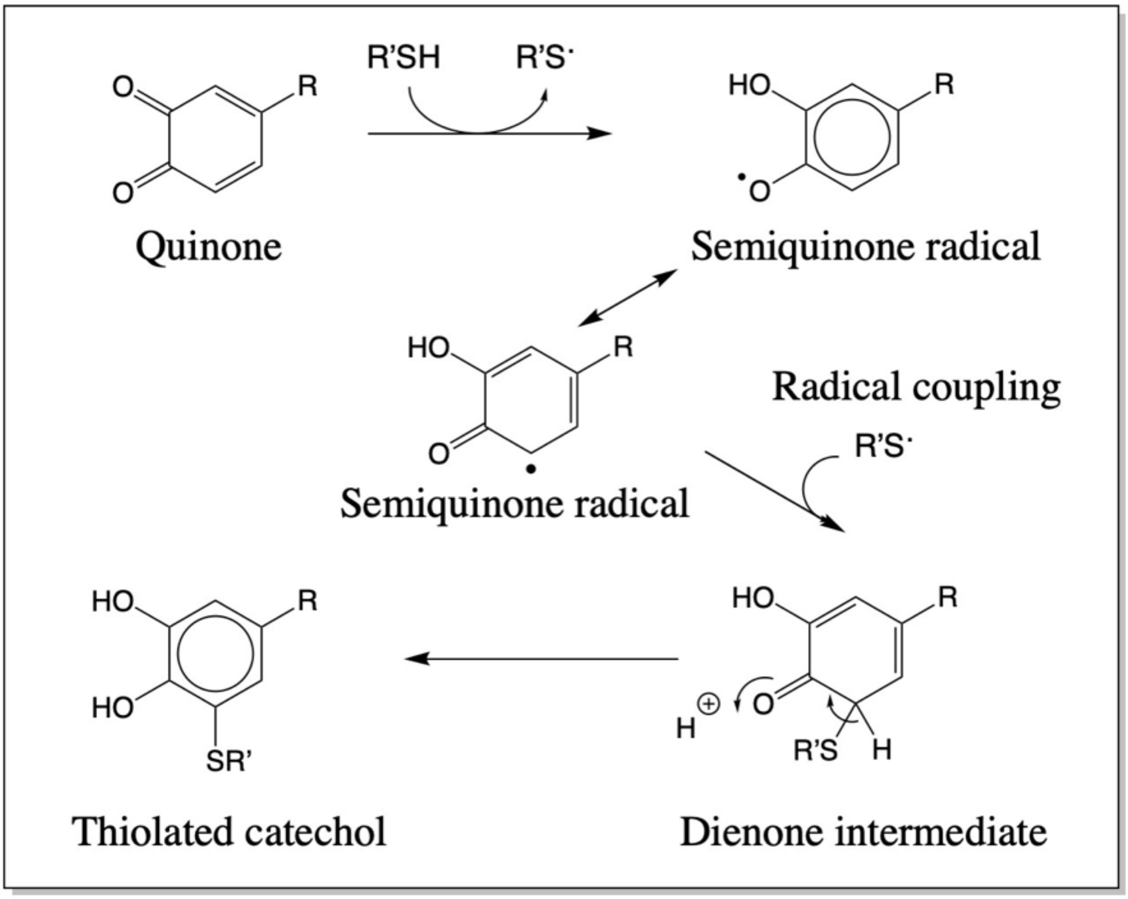

Figure S1. Alternate mechanism to account for the abnormal addition of thiols to quinone. Thiols being strong reductants, could reduce the *o*-quinone by one electron transfer to semiquinone radical. Further coupling of the semiquinone to the thinyl radical allows only Michael 1,6-addition and not the 1,4-addition observed with other typical nucleophiles.

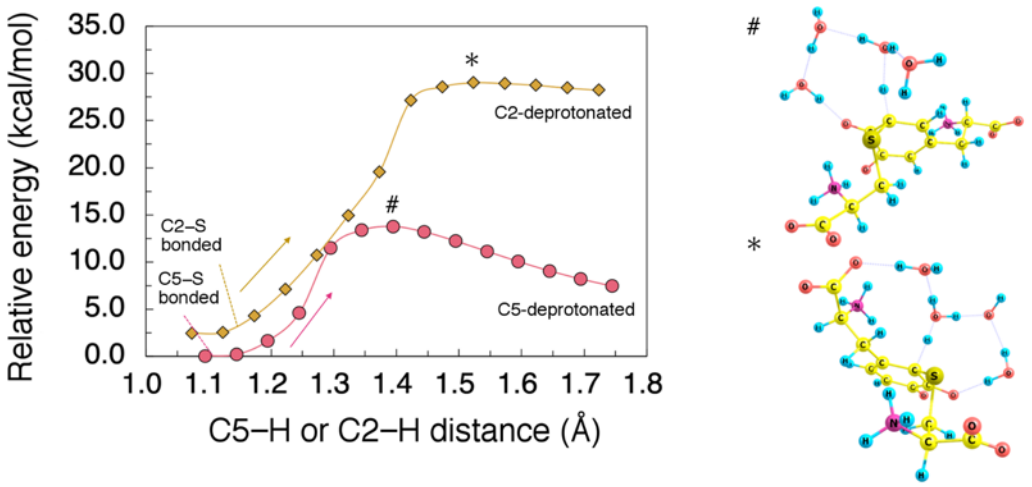

Figure S2. (Left) Minimum energy path for proton dissociation from C5 before −NH_3-to-O3 proton rearrangement, and from C2 before −NH_3-to-O4 proton rearrangement. H_2O tetramer was used as an acceptor for dissociating proton. Energies were referenced to the total energy of the initial state structure for C5-deprotonation. All geometrical parameters except for the C5−H or C2−H distance were allowed to relax. (Right) Transition state structure for C5- (#) and C2- (*) deprotonation.

Table S1. Comparison of binding energies at C5 and C3−C4 using different exchange correlation functionals

Exchange correlation functional	Binding energy (kcal/mol)	
	C5	C3−C4
B3LYP	5.8	10.9
mPW1PW91	12.7	16.8
M06-2X	20.3	25.4
CAM-B3LYP	10.1	15.9
